# The Optimal Dose of Intraoperative Dexmedetomidine for Antiemetic Effects of Post-operative Nausea and Vomiting in Patients Undergoing Elective Thoracic Surgery: A Retrospective Cohort Study

**DOI:** 10.3389/fmed.2022.891096

**Published:** 2022-04-26

**Authors:** Bing Li, Ying Zhao, Xinmin Liu, Yao Liu, Jiaqiang Zhang, Wei Zhang

**Affiliations:** ^1^Department of Anesthesiology, Henan Provincial People's Hospital, Zhengzhou, China; ^2^Department of Anesthesiology, People's Hospital of Zhengzhou University, Zhengzhou, China; ^3^Department of Anesthesiology, People's Hospital of Zhengzhou, Zhengzhou, China

**Keywords:** post-operative nausea and vomiting, dexmedetomidine, thoracic surgery, retrospective cohort, optimal dose

## Abstract

**Background:**

Dexmedetomidine (DEX) administration decreases post-operative nausea and vomiting (PONV), but it is a lack of large-scale retrospective cohort study and is unclear whether there is a dose-relationship and optimal dose for antiemetic effects between DEX and PONV. We performed a large-scale retrospective cohort study to explore the optimal dose of intraoperative DEX for antiemetic effects of PONV.

**Methods:**

A total of 5,310 patients aged ≥18 who underwent elective thoracic surgery from January 2016 to March 2020 under total intravenous anesthesia (TIVA) or combined intravenous and inhalation anesthesia in Henan Provincial People's Hospital. Patients were divided into two groups, those who received DEX intraoperatively and those who did not receive DEX. Patients who received DEX after surgery were excluded. Our primary outcomes were the association, the dose-response relationship, and the optimal dose for antiemetic effects between intraoperative DEX and PONV.

**Results:**

Among the 3,878 patients enrolled, 2,553 patients received DEX and 1,325 patients did not receive DEX. The incidence of PONV in patients who received DEX was 21.3%, and the incidence of PONV in patients who did not receive DEX was 46.5% (*P* = 0.001). After the matched-pairs cohort consisted of 1,325 patients, the incidence of PONV in patients who received DEX was 23.6%, and the incidence of PONV in patients who did not receive DEX was 46.5% (*P* = 0.001). We analyzed three different models after propensity matching to validate the stability of the prediction model between intraoperative DEX and PONV. A dose-response relationship between intraoperative DEX and PONV was observed. The optimal dose range of intraoperative DEX for antiemetic effects of PONV is 50–100 μg in elective thoracic surgery.

**Conclusions:**

Intraoperative DEX was associated with a decreased incidence of PONV in the large-scale retrospective cohort study. A dose-response relationship between intraoperative DEX and PONV was observed. The optimal dose range of intraoperative DEX for antiemetic effects of PONV is 50–100 μg in elective thoracic surgery.

## Introduction

Post-operative nausea and vomiting (PONV) include any nausea, retching or vomiting that occurs during the first 24 post-operative h (1). Vomiting can cause electrolyte imbalance and aggravate pain, even delaying discharge (2). Patients undergoing thoracic surgery experience severe pain after operation when the consumption of analgesic morphine is high, and the use of morphine is associated with nausea, vomiting, sedation and respiratory depression during acute morphine therapy (3, 4).

Fortunately, according to the fourth consensus guideline for post-operative nausea and vomiting management (5), many recommended strategies for routinely reducing the baseline risk of PONV are pointed out, including that perioperative dexmedetomidine (DEX) (evidence A1) (6). DEX 1 μg/kg before skin incision reduced the incidence of PONV after laparoscopic cholecystectomy, and PONV benefits were confirmed when DEX was added to an IV sufentanil-ondansetron PCA after thoracotomy.

However, in terms of the effect of DEX on PONV, several aspects remain unclear: (1) It is a lack of large sample size retrospective cohort study. (2) It is unclear whether there is a dose-relationship between DEX and PONV. (3) It is unclear about optimal dose of DEX for antiemetic effects.

Therefore, we hypothesized that a dose-response relationship between intraoperative DEX and PONV in elective thoracic surgery was existed. We conducted a retrospective cohort study to test this hypothesis and to explore the optimal dose of intraoperative DEX on PONV.

## Methods

### Overall Design and Data Source

This was a retrospective cohort study based on the Henan Provincial People's Hospital of China. In preparing this article, the Strengthening the Reporting of Observational Studies in Epidemiology (STROBE) checklist for cohort studies was cited. The STROBE checklist for cohort studies was referenced when preparing the article. Study design, outcome variables, and analysis plan were identified before performing the data analysis. The main page, medical record and anesthesia record sheet of each in-hospital patient was collected by Information Center Department of Henan Provincial People's Hospital and a uniform data collection system was applied. The data was obtained from an electronic medical record and collected after the fact. Anonymous data about patients' basic information, clinical diagnosis using International Statistical Classification of Diseases and Related Health Problems (10th revision) codes, surgery-relevant information, and intraoperative DEX were transferred to a specific data management institution.

### Study Population

We analyzed the data of all adult (age ≥18 yr) patients who underwent elective thoracic surgery under total intravenous anesthesia (TIVA) or combined intravenous and inhalation anesthesia between January 2016 and March 2020. Patients were excluded for the following reasons: (i) data on the classification of regional anesthesia were missing; (ii) data on nausea and vomiting in the first 24 h after surgery were not recorded; (iii) DEX was used after surgery; (iv) the patient went to the Intensive Care Unit (ICU) after surgery; (v) more than 20% of patient indicators were missing; (vi) the patient had a history of alcohol, analgesic or other drug abuse and addiction; (vii) the patient had unstable angina pectoris and myocardial infarction occurring within 3 months and New York Heart Association (NYHA) grade ≥3; and (viii) the patient had severe cardiovascular and cerebrovascular diseases. For patients who had more than one thoracic surgery during the study period, only the first thoracic surgery was included.

### Variables

Variables that may have an association with PONV were selected based on a literature review. Risk factors for PONV in adults included age, non-smoking, history of PONV or motion sickness, volatile anesthesia, risk surgery, female and post-operative opioid analgesics. Patients with completed data regarding age, sex, education, weight, smoking history, drinking history, American Society of Anesthesiologists (ASA) physical status, medical history (hypertension, diabetes mellitus, previous non-thoracic surgery, cerebral vascular and heart diseases and immune system diseases, coagulation dysfunction, History of PONV and Motion sickness), anesthesia method (TIVA and Combined intravenous and inhalation anesthesia), regional anesthesia, intraoperative dexamethasone, sufentanil and prophylactic antiemetics (5HT-3 antagonists), and surgical characteristics (surgical method, type and time), vascular drugs, bradycardia, hypotension, total infusion volume, red blood cell (RBC) transfusion, plasma transfusion, amount of bleeding, urine volume, length of stay (LOS) in the post-anesthesia care unit (PACU), patient controlled intravenous analgesia (PCIA), moderate-to-severe (MOS) pain at rest, moderate-to-severe (MOS) pain during activity, use of medication in PCIA, post-operative salvage opioid analgesics, rescue medication (5HT-3 antagonists) and PONV during the first post-operative 24 h were included in the study.

### End Points and Confounders

Nausea and vomiting are two different phenomena; they usually coexist in a patient, post-operative nausea (PON) or post-operative vomiting (POV) notably occur in parallel to PONV. Therefore, we regarded the PONV variables as a substitute for any PON, POV or retching in the trials. The most commonly used time interval to measure the role of antiemetics is 24 h post-operatively (7). We could get the occurrence and frequency of PONV within 24 h after operation. However, we could not distinguish the degree of PON and POV in our retrospective. The primary end point in our study was the incidence of PONV during the first post-operative 24 h. Secondary end points were the dose-response relationship and the optimal dose of DEX and PONV.

Baseline factors thought to have relationships with PONV were regarded as potential confounders for the analysis. Based on clinical experiences and previous studies, we adjusted for the potential confounding effects of age, sex, surgery type, surgery time, regional anesthesia, patient controlled intravenous analgesia (PCIA), education, smoking history and intraoperative sufentanil. All information concerning potential confounders was retrieved from the medical records.

### Statistical Analysis

Baseline data were stratified by categorizing the study population into two groups, dexmedetomidine and non-dexmedetomidine, according to whether dexmedetomidine was used during the operation. Continuous variables of each group are presented as the mean standard deviation (if the data are normal) or quartile, and the categorical variables are expressed as absolute values and percentages. Analysis of variance was used to compare continuous variables. Categorical variables were analyzed by the chi-squared test. A 2-tailed p <0.05 was established as the threshold of statistical significance. We did not adjust for the probability of type I errors; hence, findings concerning secondary outcome was only considered exploratory. Data analysis was performed with R packages (R version 3.4.4).

As this was a retrospective database study, the number of eligible patients was fixed; hence, we estimated the statistical power instead of calculating the sample size. And we used propensity-score matching to exclude systematic bias. Patients were matched using 1:1 nearest-neighbor matching with a caliper size of 0.05 on a propensity score scale. To control for any residual confounding by covariates with a standardized difference >10% after matching, we included these variables as adjustment for a priori selected risk factors for PONV in the multivariable logistic regression models to analyze the association between exposure and outcome. To test the robustness of our main findings, we conducted an a priori–defined sensitivity analysis, as stated above, three analysis models were devised: “Model 1” was a crude model; “Model 2” was adjusted for age and sex; and “Model 3” included age, sex, surgery type, surgery time, anesthesia method, regional anesthesia, PCIA, education, smoking history and intraoperative sufentanil as the adjustment variables.

The associations between the different doses of dexmedetomidine and PONV were analyzed to determine whether a dose-response relationship exists, in which patients with no dexmedetomidine were excluded. Bonferroni's correction was used, and 99% confidence interval (CI) was calculated in the analysis of the dose-response relationship to adjust the type I error in the multiple comparisons. Different doses of dexmedetomidine were tested to determine whether the dose-response relationship was statistically significant using the Mann-Kendall method.

## Results

**Figure 1:**
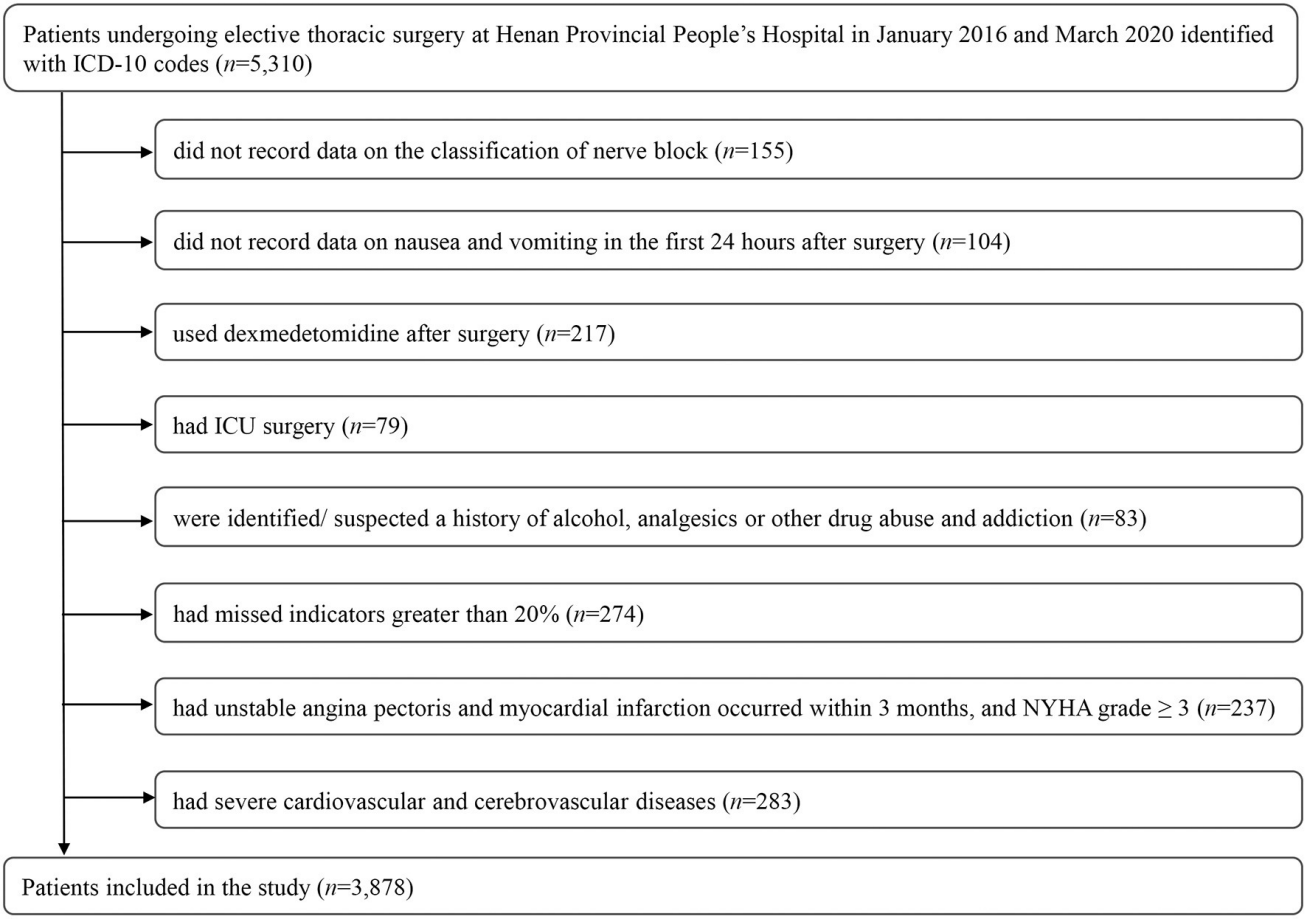
Flow chart for final patient selection in this study. DEX, dexmedetomidine; ICU, Intensive Care Unit; NYHA, New York Heart Association.

Of the 5,310 patients undergoing elective thoracic surgery identified in our database, 3,878 were eligible for inclusion (Figure 1).

### Baseline Characteristics

Among the 3,878 patients enrolled, 2,553 patients received DEX and 1,325 patients did not receive DEX. The incidence of PONV in patients who received DEX was 21.3%, and the incidence of PONV in patients who did not receive DEX was 46.5% (*P* = 0.001). We used a propensity-score matched-pairs analysis of the cohort to evaluate the adjusted association between DEX and PONV. After the matched-pairs cohort consisted of 1,325 patients, the incidence of PONV in patients who received DEX was 23.6%, and the incidence of PONV in patients who did not receive DEX was 46.5% (*P* = 0.001). There were significant differences between the groups in terms of a history of coagulation dysfunction, history of PONV and motion sickness (Table 1). There were significant differences between the groups in terms of anesthesia method, regional anesthesia, surgery type, hypotension and Urine volume (Table 2). There were significant differences between the groups in terms of PCIA, Pain during activity (MOS), use of medication in PCIA, post-operative salvage opioid analgesics, rescue medication (5HT-3 antagonists) and PONV (Table 3). We analyzed three different models after propensity matching, including Model 1 (OR = 0.497, 95% CI, 0.314–0.77; *P* = 0.002), Model 2 (OR = 0.485, 95% CI, 0.305–0.755; *P* = 0.002), and Model 3 (OR = 0.489, 95% CI, 0.305–0.768; *P* = 0.002), to validate the stability of the prediction model between DEX and PONV (Table 4).

**Table 1:** Patients characteristics.

**Items**	**Before matched**				**After matched**			
	**Without DEX** ***n* = 1,325**	**DEX** ***n* = 2,553**	***P***	***SMD***	**Without DEX** ***n* = 1,325**	**DEX** ***n* = 1,325**	***P***	***SMD***
Weight (kg)	65 (57 to 72)	65 (58 to 73)	0.019	0.076	65 (58 to 73)	65 (58 to 73)	0.768	0.009
Age (year)	57 (47 to 66)	57 (49 to 66)	0.254	0.076	57 (47 to 66)	57 (49 to 66)	0.269	0.042
Sex (male)	779 (58.8)	1,583 (62)	0.064	0.064	779 (58.8)	800 (60)	0.842	0.008
ASA physical status			<0.001	0.154			0.945	0.022
I	152 (11.5)	181 (7.1)			152 (11.5)	94 (7.1)		
II	1,016 (76.7)	2,068 (81)			1,016 (76.7)	1,073 (81)		
III	152 (11.4)	296 (11.6)			152 (11.4)	154 (11.6)		
IV	5 (0.4)	8 (0.3)			5 (0.4)	4 (0.3)		
Education			0.18	0.074			0.776	0.085
Bachelor or above	252 (19)	457 (17.9)			252 (19)	237 (17.9)		
Middle school	533 (40.2)	1,001 (39.2)			533 (40.2)	519 (39.2)		
Primary school	486 (36.7)	955 (37.4)			486 (36.7)	496 (37.4)		
Illiteracy	54 (4.1)	140 (5.5)			54 (4.1)	73 (5.5)		
Smoking history (yes)	491 (37.1)	983 (38.5)	0.414	0.029	491 (37.1)	510 (38.5)	0.842	0.043
Drinking history (yes)	443 (33.4)	888 (34.8)	0.409	0.029	443 (33.4)	461 (34.8)	0.871	0.027
History of non-thoracic surgery (yes)	496 (37.4)	978 (38.3)	0.626	0.018	496 (37.4)	507 (38.3)	0.749	0.039
Cerebral vascular disease (yes)	85 (6.4)	184 (7.2)	0.342	0.034	85 (6.4)	96 (7.2)	0.882	0.015
History of hypertension (yes)	282 (21.3)	597 (23.4)	0.14	0.051	282 (21.3)	310 (23.4)	0.819	0.014
Diabetes History (yes)	139 (10.5)	248 (9.7)	0.47	0.027	139 (10.5)	128 (9.7)	0.278	0.045
History of heart disease (yes)	85 (6.4)	245 (9.6)	0.001	0.117	85 (6.4)	87 (6.6)	0.688	0.016
History of immune system (yes)	9 (0.7)	8 (0.3)	0.121	0.061	9 (0.7)	8 (0.6)	0.117	0.070
History of coagulation dysfunction (yes)	24 (1.8)	102 (4)	< .001	0.131	24 (1.8)	53 (4)	< .001	0.090
History of PONV	49 (3.7)	71 (2.8)	0.032	0.155	49 (3.7)	31 (2.4)	0.019	0.138
Motion sickness	268 (20.3)	385 (15.1)	0.007	0.113	268 (20.3)	115 (8.7)	0.008	0.082

*Data are presented as mean (SD) or n (%) or median.*

*SD, standard deviation; DEX, dexmedetomidine; ASA, American Society of Anesthesiologists; PONV, Post-operative nausea and vomiting*.

**Table 2:** Baseline data of intraoperative patients.

**Items**	**Before matched**				**After matched**			
	**Without DEX** ***n* = 1,325**	**DEX** ***n* = 2,553**	***P***	***SMD***	**Without DEX** ***n* = 1,325**	**DEX** ***n* = 1,325**	***P***	***SMD***
Anesthesia method			0.037	0.122			0.011	0.100
TIVA	325 (24.5)	835 (32.7)			325 (24.5)	412 (31.1)		
Combined intravenous and inhalation anesthesia	1,000 (75.5)	1,718 (67.3)			1,000 (75.5)	913 (68.9)		
Regional anesthesia			< .001	0.171			0.007	0.128
TPVB	966 (72.9)	2,025 (79.3)			966 (72.9)	1,051 (79.3)		
None	359 (27.1)	528 (20.7)			359 (27.1)	274 (20.7)		
Intraoperative dexamethasone (mg)	5 (4 to 6)	5 (4 to 6)	0.146	0.087	5 (4 to 6)	5 (4 to 6)	0.613	0.083
Intraoperative sufentanil (μg)	35 (30 to 40)	35 (30 to 40)	0.162	0.041	35 (30 to 40)	35 (30 to 40)	0.831	0.015
Prophylactic antiemetics (5HT-3 antagonists) (mg)	4 (3 to 5)	4 (3 to 5)	0.341	0.040	4 (3 to 5)	4 (3 to 5)	0.526	0.047
Surgical method			>.999	0.001			0.912	0.013
Open surgery	188 (14.2)	363 (14.2)			188 (14.2)	188 (14.2)		
Endoscopic surgery	1,137 (85.8)	2,190 (85.8)			1,137 (85.8)	1,137 (85.8)		
Surgery type			< .001	0.237			0.002	0.194
Lung cancer	230 (17.4)	554 (21.7)			230 (17.4)	288 (21.7)		
Lobectomy	615 (46.4)	1,136 (44.5)			615 (46.4)	589 (44.5)		
Esophageal cancer	242 (18.3)	490 (19.2)			242 (18.3)	254 (19.2)		
Mediastinal surgery	102 (7.7)	248 (9.7)			102 (7.7)	129 (9.7)		
Thoracoscopic Sympathectomy	82 (6.2)	15 (0.6)			82 (6.2)	15 (1.1)		
Other types	93 (7)	110 (4.3)			93 (7)	50 (3.8)		
Surgery time (min)	191 (135 to 255)	190 (145 to 260)	0.043	0.057	191 (135 to 255)	190 (145 to 260)	0.872	0.001
Vascular drugs (yes)	580 (43.8)	1,220 (47.8)	0.02	0.080	580 (43.8)	633 (47.8)	0.094	0.009
Bradycardia (yes)	162 (12.2)	347 (13.6)	0.232	0.042	162 (12.2)	180 (13.6)	0.527	0.009
Hypotension (yes)	440 (33.2)	661 (25.9)	< .001	0.150	440 (33.2)	343 (25.9)	< .001	0.164
Total infusion volume (ml)	1,500 (1,000 to 2,000)	1,500 (1,100 to 2,000)	0.167	0.061	1,500 (1,100 to 2,000)	1,500 (1,100 to 2,000)	0.487	0.035
RBC Transfusion (U)	1 (1 to 1)	1 (1 to 1)	0.606	0.030	1 (1 to 1)	1 (1 to 1)	0.687	0.035
Plasma Transfusion (ml)	0 (0 to 0)	0 (0 to 0)	0.697	0.015	0 (0 to 0)	0 (0 to 0)	0.224	0.017
Amount of bleeding (ml)	100 (30 to 100)	100 (50 to 150)	<0.001	0.018	100 (30 to 100)	100 (50 to 150)	0.003	0.002
Urine volume (ml)	350 (200 to 600)	400 (200 to 600)	0.001	0.107	350 (200 to 600)	360 (200 to 600)	0.028	0.055

*Data are presented as mean (SD) or n (%) or median.*

*SD, standard deviation; DEX, dexmedetomidine; TIVA, Total intravenous anesthesia; TPVB, thoracic paravertebral regional anesthesia; LOS, Length of stay; PACU, Post Anesthesia Care Unit; RBC, red blood cell*.

**Table 3:** Patients data within 24 h after operation.

**Items**	**Before matched**				**After matched**			
	**Without DEX** ***n* = 1,325**	**DEX** ***n* = 2,553**	***P***	***SMD***	**Without DEX** ***n* = 1,325**	**DEX** ***n* = 1,325**	***P***	***SMD***
LOS in PACU (min)	70 (55 to 95)	70 (55 to 95)	0.054	0.051	70 (55 to 95)	70 (55 to 95)	0.071	0.028
PCIA (yes)	1236 (93.3)	2231 (87.4)	< .001	0.199	1236 (93.3)	1140 (86.0)	< .001	0.146
Pain at rest (MOS)	85 (6.4)	146 (5.7)	0.412	0.035	85 (6.4)	80 (6.1)	0.114	0.035
Pain during activity (MOS)	207 (15.6)	301 (11.8)	0.002	0.103	207 (15.6)	160 (12.1)	0.002	0.084
Use of medication in PCIA (μg)	206 (16.4)	199 (8.3)	0.009	0.171	206 (16.4)	133 (10.1)	0.023	0.098
Postoperative salvage opioid analgesics (μg)	167 (12.7)	125 (7.5)	0.018	0.107	167 (12.7)	112 (8.5)	0.033	0.055
Rescue medication (5HT-3 antagonists) (mg)	413 (31.2)	398 (15.6)	0.011	0.117	413 (31.2)	246 (18.6)	0.006	0.016
PONV (yes)	616 (46.5)	544 (21.3)	0.001	0.122	616 (46.5)	312 (23.6)	0.003	0.100

*Data are presented as mean (SD) or n (%) or median.*

*SD, standard deviation; DEX, dexmedetomidine; LOS, Length of stay; PACU, Post Anesthesia Care Unit; RBC, red blood cell; PCIA, patient controlled intravenous analgesia; MOS, moderate-to-severe (VAS score >3)*.

**Table 4:** Multivariable logistic regression analysis of dexmedetomidine for PONV.

**Variable**	**OR (95% CI)**	***P***
Model 1	0.497 (0.314 to 0.77)	0.002
Model 2	0.485 (0.305 to 0.755)	0.002
Model 3	0.489 (0.305 to 0.768)	0.002

*Model 1 was a crude model.*

*Model 2 was adjusted for age and sex using multivariable logistic regression.*

*Model 3 was adjusted for age, sex, surgery type, surgery time, anesthesia method, regional anesthesia, PCIA, education, smoking history and intraoperative sufentanil using multivariable logistic regression.*

*CI, confidence interval; OR, odds ratio; PONV, Post-operative nausea and vomiting; PCIA, patient controlled intravenous analgesia*.

### Dose-Response Relationship Between DEX and PONV

**Figure 2:**
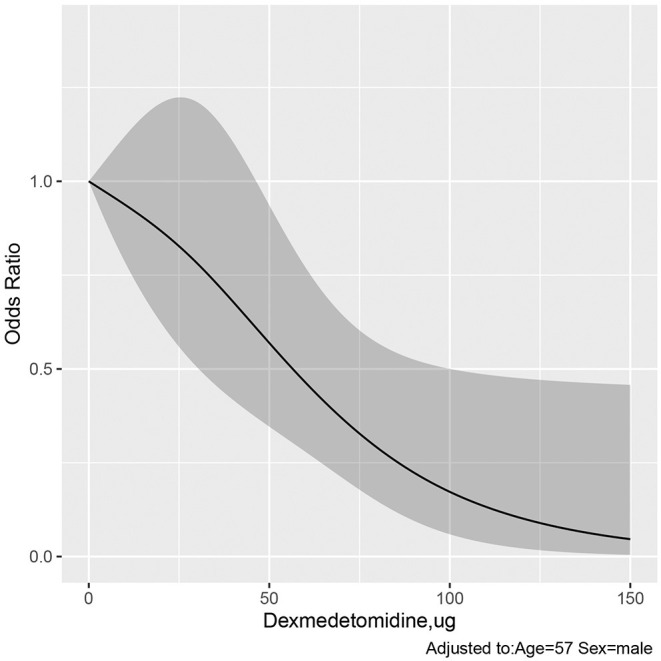
Dose–response relationship of dexmedetomidine and PONV. The ordinate is the odds ratio (0–1), and the abscissa is the dosage of DEX (0–150 μg). Error bars represent 99% confidence intervals. The larger the dose of DEX is, the lower the incidence of PONV. DEX, dexmedetomidine; PONV, Post-operative nausea and vomiting.

A dose-response relationship between DEX and PONV was observed (Figure 2). The ordinate of Figure 2 is the odds ratio (0–1), and the abscissa is the dosage of DEX (0–150 μg). As is evident, the larger the dose of DEX is, the lower the incidence of PONV.

### The Optimal Dose of DEX and PONV

The 95% upper confidence interval of OR was 1, and the critical value were 48.995 μg (OR = 0.0.604, 95% CI, 0.364–1.003) and 49.749 μg (OR = 0.595, 95% CI, 0.359–0.988) in the dose-response relationship (Figure 2). When the dose of DEX >100 μg, the OR decreases very little, and the curve is gentle (Figure 2). We analyzed three different dose range of dexmedetomidine for PONV, including 0–50 μg (OR = 0.776, 95% CI, 0.474–1.220; *P* = 0.291), 50-100 μg (OR = 0.247, 95% CI, 0.103–0.504; *P* <0.001), and 100–150 μg (OR = 0, 95% CI, 0–0; *P* = 0.988) (Table 5). Compared with 0 μg, there was only significant difference between in range of DEX in 50–100 μg. The optimal dose range of intraoperative DEX for antiemetic effects of PONV is 50–100 μg.

**Table 5:** Multivariable logistic regression analysis of different dose range of dexmedetomidine for PONV.

**Variable**	**OR (95% CI)**	***P***
0–50 μg	0.776 (0.474 to 1.220)	0.291
50–100 μg	0.247 (0.103 to 0.504)	<0.001
100–150 μg	0 (0 to 0)	0.988

*CI, confidence interval; OR, odds ratio; PONV, Post-operative nausea and vomiting*.

## Discussion

In this study, we reported three main findings: first, intraoperative DEX can reduce the incidence of PONV in patients undergoing thoracic surgery; second, a dose-response relationship between intraoperative DEX and PONV was observed; third, the optimal dose range of intraoperative DEX for antiemetic effects of PONV is 50–100 μg.

Previous small sample prospective studies have shown that perioperative DEX can reduce the incidence of PONV (7–9). Some meta-analyses demonstrated that intraoperative DEX significantly lowered post-operative pain scores and opioid consumption, which could lead to a reduced opioid-related adverse events, including PONV (6, 10). These studies focused on the specific high-risk factors for PONV, especially in women (breast and gynecological surgery) and gastrointestinal surgery. Clear risk factors independently predicting PONV included female sex, post-operative opioid treatment, prior history of motion sickness and/or PONV, and non-smokers, which can increase the risk by 20% (11). Other risk factors for PONV also included preanesthetic medication, anesthetic techniques, and post-operative pain management (12). By reviewing 4 years of patients receiving thoracic surgery in Henan Provincial People's Hospital, including esophageal surgery, lung surgery, mediastinal surgery and so on, we can further determine the relationship between intraoperative DEX and PONV. We analyzed three different models after propensity score matching and showed that perioperative DEX could reduce the incidence of PONV, further supporting that this result was very stable, and this is consistent with previous research results. To our knowledge, this is the first large-scale retrospective cohort study of intraoperative DEX and PONV in elective thoracic surgery.

The reasons why DEX could prevent PONV may be as follows: (i) Intraoperative DEX significantly lowered the demand for opioids and inhalation anesthesia during and after operation, which could help to reduce opioid-related adverse events, including PONV (13). (ii) Intraoperative DEX decreases noradrenergic activity as a result of binding to alpha-2 presynaptic inhibitory adrenoreceptors in the locus coeruleus, which may result in an antiemetic effect (14). (iii) It may be related to reducing sympathetic outflow and total catecholamine release by DEX, while high sympathetic tone and catecholamine release may trigger PONV (12).

Although some prospective studies with small sample sizes have shown that a 0.5 or 1.0 μg/kg bolus infusion could effectively decrease the incidence of PONV (7–9, 15, 16), there have been few studies on other doses, and it is not clear whether there was a dose-dependent antiemetic effect. The optimal dose of DEX for achieving antiemetic effects has not been well-documented. On the basis of the above, we explored the dose-effect relationship between DEX and PONV according to the data in our study. The intraoperative dosage of DEX ranged from 0 μg to 150 μg, and with the increase in perioperative dexmedetomidine dose, the incidence of PONV decreased. This beneficial dose-response relationship may be explained by the possible mechanism of DEX reducing the incidence of PONV.

It should be noted that bradycardia and hypotension are the most common adverse events associated with high doses of DEX, which were closely related to the rate of infusion and total dosage. Thus, when determining the optimal dose of DEX for PONV, the potential increased risk of significant hypotension and bradycardia should be balanced against optimal anti-PONV effects. We found a significant dose-response relationship between intraoperative DEX and PONV, but the range of intraoperative DEX is too extensive in Figure 2. When the 95% upper confidence interval of OR is just <1 in the dose-response relationship, the corresponding dose of DEX is 49.749 μg, indicating that some patients did not benefit from the DEX in terms of PONV when the DEX is <49.749 μg. When the dose of DEX was >100 μg, the OR value decreased very smoothly. This suggests that the benefit from DEX becomes smaller, at the same time, higher cardiovascular risk have to be considered very carefully. Meanwhile, we analyzed three different dose range of intraoperative DEX for antiemetic effects of PONV and showed that there was significant difference only when the dose of DEX was 50–100 μg. Based on our results, the optimal dose range of intraoperative DEX for antiemetic effects of PONV is 50–100 μg in elective thoracic surgery.

There were several limitations to this observational study, including (most notably) its retrospective nature, which prevented us from obtaining clinical details from decision-makers. First, the dose of intraoperative DEX was not reported per kilogram of body weight in our study, but we obtained the dose-response relationship between intraoperative DEX and PONV, and explored the optimal dose of intraoperative DEX which included different doses of intraoperative DEX for antiemetic effects of PONV. Second, previous studies have shown that intraoperative inhaled anesthetic dosage directly affected the frequency and degree of PONV. Due to the defects of retrospective study, we were unable to obtain the intraoperative inhaled anesthetic dosage. However, anesthesia methods did not affect the results of DEX for antiemetic effects after multivariate regression analysis. Third, PON and POV usually coexist in a patient, so we did not distinguish the two variables. The degree of PONV was not identified. Whether intraoperative dexmedetomidine can decrease the degree of PONV also requires further research.

In conclusion, intraoperative DEX was found to be significantly associated with a decreased incidence of PONV in a retrospective cohort study. We also observed a dose-response relationship: the greater the dose of intraoperative DEX is, the lower the incidence of PONV. The optimal dose range of intraoperative DEX for antiemetic effects of PONV is 50–00 μg in elective thoracic surgery.

## Data Availability Statement

The original contributions presented in the study are included in the article/supplementary material, further inquiries can be directed to the corresponding author/s.

## Ethics Statement

The studies involving human participants were reviewed and approved by the Ethical Committee of Henan Provincial People's Hospital, No. 7, Weiwu Road, Zhengzhou, Henan, China. Written informed consent for participation was not required for this study in accordance with the national legislation and the institutional requirements. Written informed consent was not obtained from the individual(s) for the publication of any potentially identifiable images or data included in this article.

## Author Contributions

BL and YZ: wrote the manuscript. BL and XL conducted bioinformatics analysis, analyzed the data, and drew diagrams. YL: supervision. JZ and WZ made a lot of contributions to the design of the research, conducted data analysis, graph generation, and wrote the manuscript. All authors contributed to the refinement of the study protocol and approved the final manuscript.

## Funding

This study was supported by grants from the Medical Science and Technology Research Plan Joint Construction Project of Henan Province (LHGJ20200059).

## Conflict of Interest

The authors declare that the research was conducted in the absence of any commercial or financial relationships that could be construed as a potential conflict of interest.

## Publisher's Note

All claims expressed in this article are solely those of the authors and do not necessarily represent those of their affiliated organizations, or those of the publisher, the editors and the reviewers. Any product that may be evaluated in this article, or claim that may be made by its manufacturer, is not guaranteed or endorsed by the publisher.
